# Military, Medical, and Surgical Essays

**Published:** 1864-10

**Authors:** 


MILITARY, MEDICAL, AND SURGICAL ESSAYS. Prepared for the United States Sanitary Commission. Edited by WM. A. HAMMOND, M.D., Surgeon-General U.S.A. Philadelphia; J. B. LIPPINCOTT & Co. 1864.

This is a volume of 550 pages, elegantly gotten up by the publishers. Its contents are composed of seventeen essays, on the following subjects, viz.:—

I. Military Hygiene and Therapenties, by A. C. Post, M.D., and Wm. H. Van Buren, M.D., of New York.

II. Control and Prevention of Infectious Diseases, by Elisha Harris, M.D., of New York.

III. Quinine as a Prophylactic against Malarious Diseases, by Wm. H. Van Buren, M.D., of New York.

IV. Vaccination in Armies, by F. G. Smith, M.D., and Alfred Stille, M.D., of Philadelphia.

V. Rules for Preserving the Health of the Soldier, by Wm. H. Van Buren, M.D. of New York.

VI. Scurvy, by W. A. Hammond, of Washington.

VII. Miasmatic Fevers, by John T. Metcalf, M.D., of New York.

VIII. Continued Fevers, by J. Baxter Upham, M.D., of Boston.

IX. Yellow Fever, by J. T. Metcalf, M.D., of New York.

X. Pneumonia, by Austin Flint, M.D., of New York.

XI. Dysentery, by Alfred Stille, M.D., of Philadelpha.

XII. Pain and Anesthetics, by Valentine Mott, M. D., of New York.

XIII. Hemorrhage from Wounds, and the best means for arresting it, by Valentine Mott, M.D., of New York.

XIV. Treatment of Fractures in Military Surgery, by John H. Packard, M.D.

XV. Amputations, by Stephen Smith, M.D., of New York.

XVI. The Excision of Joints for Traumatic Cause, by R. M. Hodges, M.D.

XVII. Venerial Diseases, by F. J. Bumstead, M.D. of New York.

The titles to the several essays, with the names of the authors, will give the reader abundant assurance that the volume is one of much interest and value to all members of the profession, and especially to those connected with the army.

It is for sale by S. C. Griggs & Co., Lake Street, Chicago.

